# The Effect of Wrestling Training and Sauna Practice on Cortisol and Insulin Hormones

**DOI:** 10.4236/ape.2017.73024

**Published:** 2017-08-14

**Authors:** Eser Aggon, Fatih Kiyici, Izzet Ucan, Mergul Colak, Ozturk Agirbas, Anthony C. Hackney

**Affiliations:** 1 College of Physical Education and Sports, Erzincan University, Erzincan, Turkey; 2 School of Sport Sciences, Atatürk University, Erzurum, Turkey; 3 College of Physical Education and Sports, Bayburt University, Bayburt, Turkey; 4 Department of Exercise & Sport Science, University of North Carolina, Chapel Hill, NC, USA

**Keywords:** Wrestling, Sauna, Cortisol, Insulin, Body Mass

## Abstract

**Objective:**

This study aims to determine the effect of high intensity acute wrestling exercise and sauna exposure on cortisol and insulin hormone responses.

**Method:**

After health ethics committee had granted approval, the study was conducted on 14 voluntary male elite wrestlers who had no health problems. Blood measurement was taken from the wrestlers when they were rested, after wrestling exercise and after sauna exposure which was after two days of rest following the wrestling exercise. Hormone analyses were made via IMMULITE 2000 auto-analyzer. Because of the fact that data acquired in the study were not normally distributed, nonparametric statistical analysis using SPSS was applied.

**Results:**

It was determined that, after acute wrestling exercise, there was not any significant differences in the levels of cortisol and insulin. It was also concluded that after sauna practice, while there was a significant decrease (*P* < .05) in cortisol levels, there was no significant change in insulin levels. When cortisol values were compared after wrestling exercise and sauna, it was determined that value following exercise was significantly greater (*P* < .05), but insulin values did not have a significant difference.

**Conclusions:**

It was concluded that the practice of wrestling training by elite wrestlers does not lead to changes in cortisol and insulin levels, while sauna exposure did not have an effect on insulin in a short time as in wrestling exercise but caused a significant decrease in cortisol levels.

## 1. Introduction

Wrestling is an Olympic sport which has a large mass of followers and participants throughout the world. In terms of its physical demand, it involves moving difficult load and features needed for high levels of maximal strength, explosive strength, and endurance. Furthermore, these athletes must have good reactions times in order to counter movements of their opponents (Hazar, 2000) as well as highly developed elements of the anaerobic and aerobic energy systems (Akgün, 1993).

Since the wrestlers compete in weight categories, they need to take actions to keep themselves in the limits of their weight grouping. Particularly, in the official weight measurement in one day earlier from the competition, athlete may be in position of needing to experience rapid weight reduction to adjust the weight of category they will compete in. Such situations have led wrestlers to use different methods of weight reduction; such as, exercise and heat exposure and passive dehydration (heat exposure) and fluid and food restriction (Lambert & Jones, 2010).

Besides sauna is an important application that individuals use with the aim of deep physical and mental regeneration (Tsonis, 2017), it also takes place in many sportive aim training practices (Pilch, 2008; Kukkonen-Harjula & Kauppinen, 2006). Particularly, in sport group like wrestling, which is related to the weight, sometimes, athletes make sauna application to enable rapid weight reduction. Sauna application has the potential to affect the muscle circulation, and respiration system of the body, and it is known that it has effects on endocrine system. As in exercise, the state of high heat exposure develops a physiological stress in the body (Huether & McCance, 2009). Cortisol is a hormone, whose amount of release in the blood varies as a reaction to the stress state (Hackney, 2006), and has effects on the carbohydrate, protein and fat metabolism (Junqueira et al., 1993). Increase in the release of cortisol shows an increase in parallel with the increase of stress in exercise (Hackney). When the level of blood glucose rises, insulin hormone is secreted (Günay, Kara, & Cicioğlu, 2006). Insulin is a hormone, which stimulates the anabolic state in organism, and makes contribution to storing carbohydrates and lipids as well as protein synthesis; and has an important role in providing the inlet of glucose to the cell, reducing the level of blood glucose (Ersöz, 2002).

The intent of this study was been to identify the physiological stress of wrestlers emerging before and after exercise as compared to the effects of sauna as thermal a physiological stress. The aim of this study is to separately identify the effects of acute wrestling exercise (high intensity) and sauna practice on critical hormonal biomarkers.

## 2. Materıals and Methods

The study was conducted before the competition session.

### Subject:

Elite wrestler athletes (n = 14; age: 23.43 ± 4.1 years, height: 172.8 ± 4.8 cm, body mass: 74.9 ± 14.5 kg and BMI 24.9 ± 3.4 kg/m^2^) volunteered to participate in the study. All wrestlers participated representing Turkey in some ınternational tournaments like European Championships and other tournaments.

The inclusion criteria for participants were: non-smoker, no known history of cardiovascular and endocrine disease, wrestling training history > 5 years, and no intake of medication or endocrinal supplements. All participants provided informed consent.

### Experimental Protocol:

All testing sessions took place at the same time of days (16.00 – 17.00). There was an exercise (wrestling) session performed in competition style. After the participants rested for two days, then there was exposed to sauna. Their body weight and height data was measured with a precision scales in pre and post wrestling exercise sessions and post sauna.

### Wrestling Protocols:

Wrestlers warmed up as the match procedure during 30 minute. After the warm up, wrestling training was performed in match-style in three sessions for 3 × 2 minutes and the wrestlers were asked to performance at maximal levels.

### Sauna Protocols:

After the participants rested for 48 hours, they were taken into sauna sessions at 80°C – 100°C where humidity was 10% - 15%. The sauna applications consisted of 3 × 20 minute sauna exposure with 2 minute breaks at normal room temperature.

### Collection of Blood Samples:

Approximately, 10 ml blood samples were taken before exercise, after the wrestling exercise and following sauna from antecubital vein. All blood samples were drawn into EDTA-treated tubes and placed on ice until processing. Separated plasma was frozen at −80°C until later hormones analysis.

### Biochemical Assays:

All biochemical analyses done Atatürk University Medical Faculty Biochemistry Laboratorıes by expert biochemists. Hormone analyses measurement were performed on an Immulite 2000 analyzer (Diagnostic Products Corporation) using manufacturer’s reagents according to instructions.

### Statistical Analyses:

Statistical analyses were carried out using the SPSS 19. Analysis was performed to determine whether the data were normally distributed. It was determined that the data sets were not normally distributed therefore non-parametric analysis were performed. Specifically, Friedman and Wilcoxon tests were used to compare values. Statistically significant level accepted as 0.05.

## 3. Results

From Table 1, in both wrestling and sauna treatment, it is seen that the values of body mass and body mass index significantly decreased. It was also identified that cortisol values after sauna were significantly low (*P* < .05), compared to both rest state and post-acute wrestling exercise and that cortisol values of acute wrestling exercise did not show a significant change (i.e., ↑) compared to the rest state. Neither the wresting exercise nor the sauna exposure had a significant effect on insulin.

## 4. Discussion

As noted earlier, the aim of this study is to separately identify the effects of acute wrestling exercise (high intensity) and sauna practice on critical hormonal biomarkers.

It is important to first note that both the experimental treatments influenced the body weight (and hence the BMI) of the participants. Hence our exercise and sauna conditions were efficacious. That is, we identified that the values of body mass and body mass index were significantly decreased (Table 1); this significant reduction was expected based upon previous literature (Boutcher, 2011; Gutierrez et al., 2003). Both the intensity of exercise and the increased body heat by the sauna caused weight loss in the wrestlers. However, it is important to note that the magnitude of these reductions were quite small.

### 4.1. Exercise Hormonal Responses

We found no differences in cortisol and insulin levels between pre and post exercise (Table 1). These results were not unexpected and surprised us as many studies have found significant increases in cortisol levels increased in post wrestling exercise compared to resting values (Kraemer et al., 2001; Fry et al., 2011; Coelho, Keller, & Da Silva, 2010). Although, several other research groups have also reported no differences in cortisol levels between pre and post wrestling exercises (Ratamess et al., 2013; Roemmich & Sinning, 1997). It is unclear why we did not see the expected increase in cortisol following the exercise, but Daly et al. (2004), has suggested that the sampling time of cortisol following exercise is critical and strongly effect whether significant changes are detected.

There was a trend observed for a decrease at insulin levels after wrestling exercise we apply but it was not statistically significant. Roemmich & Sinning (1997) studied young wrestlers, and similar to us did not find significant differences in the insulin values before and after wrestling exercise. Kraemer et al. (2001) identified that the values of blood glucose with wrestling competition greatly increased after competition but no significant difference occurred in insulin values. However, Cicioğlu and Onay (2002) reported a significant decrease occurred in the insulin values of wrestlers after bicycle ergometer exercise. In the other studies, as a result of prolonged exercise times, it was reported that there was a decrease at insulin levels (Koistinen et al., 1998; Kowalska et al., 1999; Torjman et al., 1999; Essig et al., 2000). Perhaps even though our exercise was highly intensive it was not prolonged enough in duration to invoke a reduction in insulin.

### 4.2. Sauna Hormonal Responses

We identified that there was a significant decreased at the cortisol levels of the wrestlers after sauna practice (Table 1). The results of our study are not in agreement with the literature. For example, Iwase et al. (2013) identified that cortisol values of subjects did not vary after sauna exposure at 70°C. Pilch et al. (2008) contrastingly carried out a study in 10 females, and found that cortisol significantly increased after sauna. In addition, Pilch et al. (2013) found significant increases in the cortisol values after sauna session they applied for 15 minutes to 9 trained middle-distance runners. Although there are not many studies examining the relationship between sauna and cortisol level in the literature, the results obtained in these limited studies seem to differ from one another. As a result of our study, it is considered that unexpected fall at the cortisol level resulted from the habits of group of elite wrestlers regarding sauna practice (chronic exposure), thus, state of physiological adaptation to high ambient temperature; and beside this, that the duration of sauna session applied was more than those applied in other studies.

Furthermore, after sauna exposure there was no significant difference in insulin. The limited studies carried out to examine the relationship between insulin and sauna are in agreement with the results of our study. Lammintausta et al. (1976) and Jenssen et al. (1988) each also stated that insulin values after sauna did not vary from rest.

When the values of wrestling exercise and control values after sauna are compared, it was identified that the values of wrestling group were significantly high (*P* < .05), while there was no a significant difference in insulin values.

When compared to the effect levels of wrestling and sauna on cortisol that it was seen the effect of wrestling exercise is significantly higher than sauna (*P* < .05).

## 5. Conclusion

It was concluded that in elite wrestlers after both wrestling exercise and sauna exposure body mass and body mass index significantly decreased, but to a small magnitude. Wrestling exercise applied in competition type-model did not produce a significantly difference at the cortisol and insulin levels; while sauna exposure carried out for 3 × 20 minutes caused a significant change in cortisol levels, but it did not lead to a significant change in insulin levels. Finally, when cortisol values after wrestling exercise and sauna are compared, the values of exercise are significantly greater, while there is no significant difference in the insulin values.

## Figures and Tables

**Table 1.** Values of wrestlers’ body mass, body mass index, cortisol and insulin during rest, after exercise and sauna.

Friedman							Wilcoxon
		N	Mean Rank	Med	X^2^	P	
**Body Mass** (kg)	**Resting (1)**	14	2.57	70.95	7.840	.020*	1 – 2* (.017) 1 – 3* (.041)
	**After Exercise (2)**		1.79	70.8			
	**After Sauna (3)**		1.64	70.7			
**Body Mass Index** (kg/m^2^)	**Resting (1)**	14	2.50	24.15	7.409	.025*	1 – 2* (.019) 1 – 3* (.032)
	**After Exercise (2)**		1.89	24.1			
	**After Sauna (3)**		1.61	23.95			
**Cortisol** (mg/dl)	**Resting (1)**	14	2.36	8.95	15.571	.000*	1 – 3* (.009) 2 – 3* (.003)
	**After Exercise (2)**		2.50	11.11			
	**After Sauna (3)**		1.14	4.78			
**Insulin** (μIU/mL)	**Resting (1)**		2.00	6.49	3.571	.168	-
	**After Exercise (2)**	14	1.64	5.83			
	**After Sauna (3)**		2.36	10.1			

*: statistically significant different from baseline (*P* < .05).
